# Trends, Predictors, and Outcomes of Monitored Acute Care Unit Admissions in Older Adults: 10-Year Retrospective Analysis

**DOI:** 10.2196/80629

**Published:** 2026-02-02

**Authors:** Ludovic van Delden, Stephan von Düring, Frederic Rouyer, Anne Rossel, Christophe Marti, Sara Cereghetti, Xavier Roux, Thibaut Desmettre, Olivier Grosgurin, Christophe A Fehlmann

**Affiliations:** 1 Division of Critical Care Medicine, Department of Acute Medicine, Geneva University Hospitals, Geneva, Switzerland; 2 Department of Anaesthesiology, Clinical Pharmacology, Intensive Care and Emergency Medicine, Faculty of Medicine, University of Geneva, Geneva, Switzerland; 3 Division of Emergency Medicine, Department of Acute Medicine, Geneva University Hospitals, Rue Gabrielle Perret Gentil 4, Geneva, 1211, Switzerland, 41 795535933; 4 Division of Internal and General Medicine, Department of Internal Medicine, Geneva University Hospitals, Geneva, Switzerland; 5 Department of Medicine, Faculty of Medicine, University of Geneva, Geneva, Switzerland; 6 Division of Internal Medicine for the Elderly, Department of Rehabilitation and Geriatrics, Geneva University Hospitals, Geneva, Switzerland

**Keywords:** older patients, intensive care units, intermediate care units, critical care, hemodynamic monitoring, risk assessment

## Abstract

**Background:**

Global population aging places an increasing burden on health care systems. This is driven by multimorbidity, frailty, and polypharmacy. Older adults, particularly those aged 65 years or older, use emergency departments (EDs) more frequently and experience poorer outcomes. In this population, decisions regarding admission to monitored acute care units—intensive care units, intermediate care units, and operating rooms—are frequent and complex. While ED and intensive care unit use are well documented, data on monitored acute care units as a whole remain limited. Evidence on admission trends, patient characteristics, and outcomes in older adults is scarce.

**Objective:**

This study aimed to describe temporal trends in monitored acute care unit admissions, identify predictors of such admissions, and assess outcomes following these admissions.

**Methods:**

We conducted a retrospective cohort study using routinely collected electronic health record data. We included patients aged 65 years or older who visited the EDs of the Geneva University Hospitals, Switzerland, between 2009 and 2019. The primary outcome was admission to a monitored acute care unit. The secondary outcomes were hospital length of stay, 7-day mortality, and 1-year mortality. Logistic regression models were used to identify factors associated with monitored acute care unit admission and to assess the association between age and mortality.

**Results:**

During the 10-year period, 701,838 ED visits were recorded. Annual visits increased from 56,944 to 76,368 (+34.1%). The increase was greater among patients aged 65 years or older (+56.1%) than among younger patients (+26.5%). A total of 180,189 older patients presented to the ED. Of these, 887 (0.5%) died in the ED, 97,238 (54.0%) were discharged home, 63,025 (35.0%) were admitted to a ward, and 19,039 (10.6%) were admitted to a monitored acute care unit. Monitored acute care unit admissions increased from 1379 (10.3%) in 2009 to 2240 (11.1%) in 2019. This represented an absolute increase of 62.4% and a relative increase of 0.8%. Predictors of monitored acute care unit admission included younger age, male sex, ambulance arrival, higher triage level, being married or in a relationship, not residing in a nursing home, and French as the primary language. Among patients admitted to a monitored acute care unit, mortality was 5.8% (1105/19,039) at 7 days and 22.3% (4251/18,039) at 1 year. Older age was associated with higher 7-day mortality (adjusted odds ratio 1.55, 95% CI 1.14‐2.10) and 1-year mortality (adjusted odds ratio 1.28, 95% CI 1.08‐1.51).

**Conclusions:**

Admissions to monitored acute care units among older patients increased over time. These findings indicate a growing demand for high-level care in this population. Hospitals should adapt infrastructure and resource allocation to address the needs of an aging population.

## Introduction

The global population is aging rapidly, exerting substantial pressure on health care systems [1,2]. With increasing life expectancy, older adults present with a higher burden of chronic illnesses, polypharmacy, and biological frailty. However, only limited data exist on the use of monitored acute care units within this population.

Individuals aged between 65 and 79 years exhibit the highest prevalence of comorbidity, while those aged 80 years and older have a 30% likelihood of presenting with at least one chronic condition [3]. This demographic shift places considerable strain on health care services. In the United States, studies have shown that patients aged older than 65 years account for 15% of all emergency department (ED) admissions, with these consultations increasing by 25% over the past decade [4,5]. These patients typically present with more severe emergencies, requiring extensive hospital resources [6]. Moreover, they experience poorer outcomes following admission compared to younger patients, particularly in the intensive care unit. In-hospital mortality rates are markedly higher for older patients during the postintensive phase [7,8], with the majority of deaths occurring within 3 months after intensive care unit discharge [9]. Furthermore, survivors frequently experience a substantial decline in quality of life, with age and frailty being crucial factors that affect the recovery of functional capacity [10].

These age-associated challenges necessitate careful consideration when evaluating older patients for admission to high-level care. Understanding these aspects is essential for health care providers in developing strategies that effectively address the specific needs of older patients, ensuring that the allocation of high-level care resources is both efficient and beneficial. While extensive research exists on ED use and intensive care unit admissions in older patients, there is a notable gap in data regarding the trends in monitored acute care unit admissions over time. Furthermore, research on monitored acute care unit admissions, encompassing intensive care units, intermediate care units, and operating rooms, as a whole entity, remains limited. Finally, intermediate care units have only recently been introduced in health care systems, and their impact on patient disposition and role as acute care providers remains to be determined.

This study aimed to address this gap by analyzing ED and monitored acute care unit admissions among patients aged 65 years or older over the past decade in a Swiss tertiary care institution. Our primary objective was to describe the trends in monitored acute care unit admissions during this period. Our secondary objectives were to identify predictors of monitored acute care unit admission, describe patient outcomes following monitored acute care unit admission, and explore the association between patient age and mortality at day 7 and 1 year postadmission.

## Methods

### Study Design

This single-center retrospective cohort study used routinely collected data from patients who visited the ED between 2009 and 2019. The study was approved by the institutional ethics committee of Geneva, Switzerland, and patient consent was waived based on Article 34 of the Swiss law on human research.

### Study Setting and Population

The study was conducted at Geneva University Hospitals (HUG), the largest medical complex in Switzerland. The HUG is a tertiary care institution with 2 adult EDs located at different sites. The main ED, which operates 24/7, handles approximately 80,000 visits annually and includes a stretcher bay and a walk-in sector, providing comprehensive emergency care. The second ED, opened in 2016, is situated at a geriatric hospital and handles approximately 5000 visits annually. It operates only during daytime hours and is dedicated to patients aged older than 75 years with low-severity conditions. Although this geriatric ED is equipped with a full radiological division, it has limited access to specialty consultants, sometimes requiring the transfer of patients who need urgent specialized consultation or surgical procedures. Hospitals within the HUG network share the same computer system, and patients’ medical data are accessible in each hospital.

HUG provides specialized care across all medical fields, serving as a reference center for regional hospitals and often receiving patients from smaller centers. The main location offers 24-hour cardiac catheterization services and is the only trauma and stroke center in the region, providing immediate care for patients requiring intravenous thrombolysis, interventional radiology, or the management of life-threatening injuries.

The HUG’s critical care infrastructure includes a medical-surgical intensive care unit and several specialized intermediate care units. These intermediate care units serve as an intermediary level of care between the intensive care unit and regular wards and include a cardiac intermediate care unit, a neurological and neurosurgical intermediate care unit (with a stroke unit), a surgical intermediate care unit, and 2 medical intermediate care units (one located at the main hospital and one at the geriatric hospital).

The HUG is funded through a mixed financing model, with approximately half of the budget provided by the State of Geneva and the remainder generated from hospital revenues (including patient care and insurance reimbursements) as well as dedicated research funding from national grants and private foundations. Health care financing in Switzerland is based on mandatory basic health insurance (LAMal), which ensures equal access to care for all residents and is funded through individual premiums, complemented by regulated cost-sharing mechanisms (eg, deductibles and co-payments) and optional supplementary insurance.

### Participants

We included all adult patients (aged ≥18 y) who visited either of HUG’s EDs between January 1, 2009, and December 31, 2019. Patients were excluded if they (1) were aged younger than 65 years, (2) were transferred from another hospital or ED outside of HUG, or (3) refused the use of their data for research purposes. The age cutoff of 65 years was selected, as it is a widely recognized threshold for defining “older patients” and aligns with the retirement age in Switzerland.

### Variables and Data Sources

The primary outcome was monitored acute care unit admission, defined as an admission to an operating room (including surgical rooms, interventional radiology, or a cardiac catheterization laboratory), an intermediate care unit, or the intensive care unit. This outcome was based on patient trajectories within the institution. The secondary outcomes included hospital length of stay, mortality at day 7 after monitored acute care unit admission, and mortality at 1 year. Owing to the lack of linkage between the electronic health record and the national death registry, data were missing for patients who did not die in the hospital and were not readmitted beyond 1 year after the initial admission.

Patient variables extracted included age, sex, marital status (married or in a relationship vs single, divorced, or widowed), primary language, emergency triage level (based on the Swiss Emergency Triage Scale [11], ranging from 1 [highest priority] to 4 [lowest priority]), triage motive category, nursing home residence, and means of transportation to the ED (ambulance or nonambulance).

Data were electronically extracted from the hospital’s data warehouse by a dedicated team, which contains all information routinely gathered for clinical use in the patient’s electronic health record. These structured data can be retrieved for quality assessment or research purposes after approval from the research ethics board. Each care episode is assigned a unique number, facilitating linkage between different database subsets. There was no linkage with other databases.

### Study Size

The sample size was determined based on the study’s objective to describe trends over a decade rather than on power calculations for statistical significance. With an expected inclusion of more than 150,000 patients, the sample size was considered sufficient to perform a multivariable logistic regression analysis without the risk of overfitting, based on established methodological recommendations regarding events per variable. Previous studies have shown that logistic regression models are unlikely to be overfitted when at least 10 events per predictor variable are available and that model performance remains robust even with fewer events per variable in large datasets with stable estimates. Given the large number of observations and outcome events in this study, the risk of overfitting was therefore considered minimal [12-14].

### Statistical Analysis

Data cleaning and statistical analysis were conducted using the integrated statistical software Stata/SE (version 18; StataCorp LLC).

First, the data were cleaned and standardized. Variables were reformatted to facilitate analysis. Outcomes were constructed based on patients’ trajectories through the institution. To evaluate trends in monitored acute care unit admissions over time, we graphically computed the number of ED visits for patients aged 65 years or older and the number and type of monitored acute care unit admissions by year. The absolute and relative changes over time were reported. A linear regression model was used to evaluate trends over time and determine the statistical significance of the observed changes.

To explore predictors of monitored acute care unit admission, baseline variables were summarized using descriptive statistics for the overall population. Mean and SD (or median and IQR) were used for continuous variables, whereas frequency and proportion were used for categorical variables. Patients admitted to a monitored acute care unit were compared to those not admitted to a monitored acute care unit using standardized mean differences (SMDs). We used SMDs rather than *P* values, as these comparisons are descriptive and not inferential. An absolute SMD greater than 0.2 was considered to reflect a potentially meaningful imbalance. The association between age and monitored acute care unit admission was graphically represented using restricted cubic splines, a flexible regression method, with the number of knots determined by the Akaike information criterion and their placement based on recommended quantiles [14]. Independent predictors of monitored acute care unit admission were identified using a multivariable logistic regression model, with monitored acute care unit admission as the dependent variable. Potential predictors were selected based on previous literature or clinical relevance and included sex, age, marital status, nursing home residence, primary language, arrival by ambulance, emergency triage level, and triage motives. Relationships modeled with restricted cubic splines were presented as odds ratios (ORs), comparing older patients (75th percentile of age) to younger patients (25th percentile of age) [14]. A sensitivity analysis using age as a categorical variable was also performed.

To explore patient outcomes following monitored acute care unit admission, outcomes were summarized using descriptive statistics.

Finally, to explore the association between patient age and mortality at day 7 and 1 year following monitored acute care unit admission, restricted cubic splines were used. Subsequently, multivariable models similar to those used previously were applied.

Missing data frequency was reported for each variable. For the initial logistic regression model, a complete case analysis was performed. A subsequent analysis included variables with higher levels of missingness without imputation to assess changes in the coefficient estimates.

### Ethical Considerations

The study was conducted at HUG in accordance with the principles of Good Clinical Practice (the Declaration of Helsinki, 2002). This study was approved on July 11, 2022, by the institutional ethics committee of Geneva, Switzerland (project ID 2022‐00987). Patient consent was waived by this committee. No compensation was offered to participants Data extraction generated deidentified data used for the analysis. These data were subsequently anonymized for online publication.

## Results

### Inclusion of Patients and Characteristics

**Figure 1.**
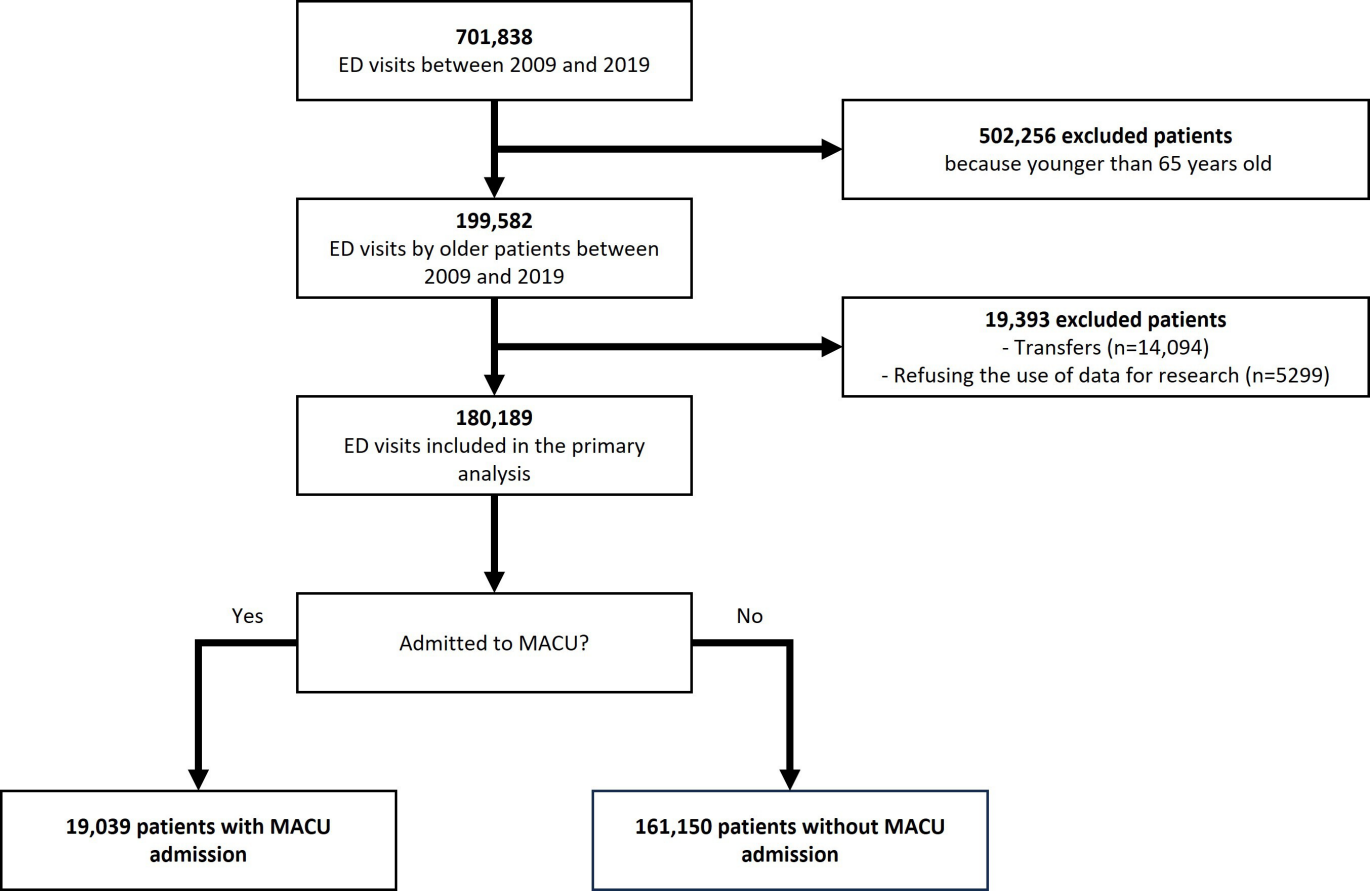
Flowchart of our retrospective cohort study conducted at the Geneva University Hospitals (HUG) over a 10-y period (2009‐2019). All adult patients (aged ≥18 y) who visited the HUG’s emergency departments (EDs) were candidates for inclusion. Patients were excluded if they (1) were aged <65 y, (2) were transferred from another hospital or ED outside of HUG, or (3) refused the use of their data for research purposes. Of the 701,838 patients in our dataset, 180,189 met our inclusion criteria, representing 90.3% of all patients aged ≥65 y admitted to the ED during the study period. Of these patients, 19,039 (10.6%) were admitted to a monitored acute care unit (MACU).

Between 2009 and 2019, a total of 701,838 ED visits were recorded at our institution (Multimedia Appendix 1). Of these, 180,189 (25.7%) patients met our inclusion criteria (Figure 1). The median age was 79 (IQR 72-86) years. Patient characteristics and the proportion of missing data are presented in Table 1.

**Table 1.** Baseline characteristics.

	Total (N=180,189)	With MACU^a^ admission (n=19,039)	Without MACU admission (n=161,150)	SMD^b^
Age (y), median (IQR)	79 (72-86)	78 (72-84)	79 (72-86)	0.120
65‐74, n (%)	61,511 (34.1)	6890 (36.1)	54,621 (33.9)	0.153
75‐84, n (%)	66,603 (37.0)	7668 (40.3)	58,935 (36.6)	—^c^
85‐94, n (%)	46,890 (26.0)	4190 (22.0)	42,700 (26.5)	—
≥95, n (%)	5185 (2.9)	291 (1.5)	4894 (3.0)	—
Sex, n (%)				0.150
Male	82,877 (46.0)	10,027 (52.7)	72,850 (45.2)	
Female	97,312 (54.0)	9012 (47.3)	88,300 (54.8)	
Marital status, n (%)				0.152
Married or in a relationship	79,499 (44.1)	9661 (50.7)	69,838 (43.3)	
Single, divorced, or widowed	100,435 (55.7)	9297 (48.8)	91,138 (56.6)	
Missing	255 (0.1)	81 (0.4)	174 (0.1)	
Nursing home resident, n (%)				0.058
No	164,454 (91.3)	17,552 (92.2)	146,902 (91.2)	
Yes	11,967 (6.6)	1027 (5.4)	10,940 (6.8)	
Missing	3768 (2.1)	460 (2.4)	3308 (2.1)	
Primary language, n (%)				0.123
French	56,788 (31.5)	6408 (34.0)	50,308 (31.2)	
Italian	5958 (3.3)	555 (2.9)	5403 (3.4)	
Spanish	3290 (1.8)	254 (1.3)	3036 (1.9)	
German	2069 (1.1)	233 (1.2)	1836 (1.1)	
English	1552 (0.9)	151 (0.8)	1401 (0.9)	
Portuguese	1355 (0.8)	111 (0.6)	1244 (0.8)	
Others	3821 (2.1)	326 (1.7)	3495 (2.2)	
Missing	105,536 (58.5)	10,929 (57.4)	64,427 (58.6)	
Arrival by ambulance, n (%)				0.478
No	66,881 (37.1)	3967 (20.8)	62,914 (39.0)	
Yes	91,829 (51.0)	13,572 (71.3)	78,257 (48.6)	
Missing	21,479 (12.0)	1500 (7.9)	19,979 (12.4)	
Triage scale, n (%)				1.152
1: vital emergency	20,921 (11.6)	8290 (43.5)	12,631 (7.8)	
2: urgent	65,305 (36.2)	7886 (41.4)	57,419 (35.6)	
3: Mildly urgent	89,840 (49.9)	2786 (14.6)	87,054 (54.0)	
4: nonurgent	3788 (2.1)	31 (0.2)	3757 (2.3)	
Triage motive category, n (%)				0.759
Cardiology-pneumology	48,348 (26.8)	7550 (39.7)	40,798 (25.3)	
Neurology-psychiatry	29,972 (16.6)	6318 (33.2)	23,654 (14.7)	
Traumatology	30,202 (16.8)	1598 (8.4)	28,604 (17.8)	
Digestive-OB/GYN^d^	17,731 (9.8)	1392 (7.3)	16,339 (10.1)	
Urology-nephrology	8285 (4.6)	263 (1.4)	8022 (5.0)	
Rheumatology	6793 (3.8)	105 (0.6)	6688 (4.2)	
Infectious disease	5813 (3.2)	435 (2.3)	5378 (3.3)	
Dermatology	4580 (2.5)	134 (0.7)	4446 (2.8)	
ENT^e^	3883 (2.2)	100 (0.5)	3783 (2.3)	
Others	24,263 (13.5)	1107 (5.8)	23,156 (14.4)	
Missing	319 (0.2)	37 (0.2)	282 (0.2)	

a MACU: monitored acute care unit.

b SMD: standardized mean difference.

c Not applicable.

d OB/GYN: obstetrics and gynecology.

e ENT: ear, nose, and throat.

### Monitored Acute Care Unit Admissions and Evolution Over Time

Over the study period, 887 (0.5%) patients died in the ED, 97,238 (54.0%) patients were discharged home, 63,025 (35.0%) patients were admitted to a ward, and 19,039 (10.6%) patients were admitted to a monitored acute care unit. Of these, 4499 (23.6%) patients were admitted to the intensive care unit, 10,835 (56.9%) patients were admitted to an intermediate care unit, and 3705 (19.5%) patients were admitted directly to an operating room (Table 2).

**Table 2.** Patients’ disposition from the emergency department by age categories.

	Total (N=180,189), n (%)	65‐74 y (n=61,511), n (%)	75‐84 y (n=66,603), n (%)	85‐94 y (n=46,890), n (%)	≥95 y (n=5185), n (%)
MACU^a^	19,039 (10.6)	6890 (11.2)	7668 (11.5)	4190 (8.9)	291 (5.6)
Intensive care unit	4499 (23.6)	1958 (28.4)	1917 (25.0)	611 (14.6)	13 (4.5)
Intermediate care unit	10,835 (56.9)	3508 (50.9)	4339 (56.6)	2788 (66.5)	200 (68.7)
Operating room	3705 (19.5)	1424 (20.7)	1412 (18.4)	791 (18.9)	78 (26.8)
Ward	63,025 (35.0)	17,917 (29.1)	23,999 (36.0)	18,953 (40.4)	2156 (41.6)
Discharged	97,238 (54.0)	36,534 (59.4)	34,608 (52.0)	23,411 (49.9)	2685 (51.8)
Died in the emergency department	887 (0.5)	170 (0.3)	328 (0.5)	336 (0.7)	53 (1.0)

a MACU: monitored acute care unit.

**Figure 2.**
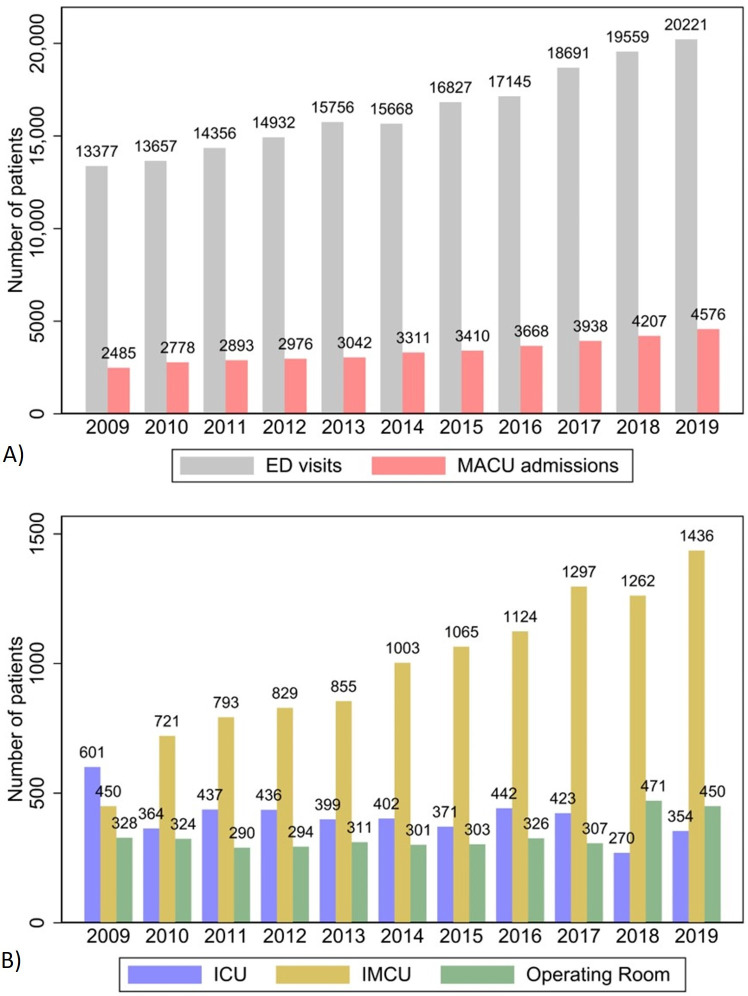
(A) Trends in emergency department (ED) visits and monitored acute care unit (MACU) admissions for patients aged ≥65 years and (B) distribution of MACU admissions to different types of MACUs over time. Over the 10-year study period (2009‐2019), ED visits at the Geneva University Hospitals from patients aged ≥65 years increased by 56.1%, from 14,705 to 22,955. MACU admissions increased by 62.4%, from 1379 to 2240. The increase in MACU admissions primarily involved intermediate care units (IMCUs), from 450 of 1379 (32.6%) in 2009 to 1436 of 2240 (64.1%) in 2019. ICU: intensive care unit.

Over the 10-year study period, monitored acute care unit admissions increased by 62.4% in absolute terms (from 1379 in 2009 to 2240 in 2019) and by 0.8% in relative terms (from 10.3% in 2009 to 11.1% in 2019, *P*=.02; Figure 2A). This increase was mainly driven by a rise in intermediate care unit admissions, from 450 of 1379 (32.6%) in 2009 to 1436 of 2240 (64.1%) in 2019. In contrast, operating room admissions remained stable, from 328 of 1379 (23.8%) to 450 of 2240 (20.1%). Intensive care unit admissions decreased, from 601 of 1379 (43.6%) to 354 of 2240 (15.8%; Figure 2B). Notably, while the proportion of patients who died in the ED remained stable, the proportion of patients admitted to hospital wards increased over the 10-year period, from 4026 of 13,377 (30%) in 2009 to 7996 of 19,990 (40%) in 2019.

### Predictors for Monitored Acute Care Unit Admission

**Figure 3.**
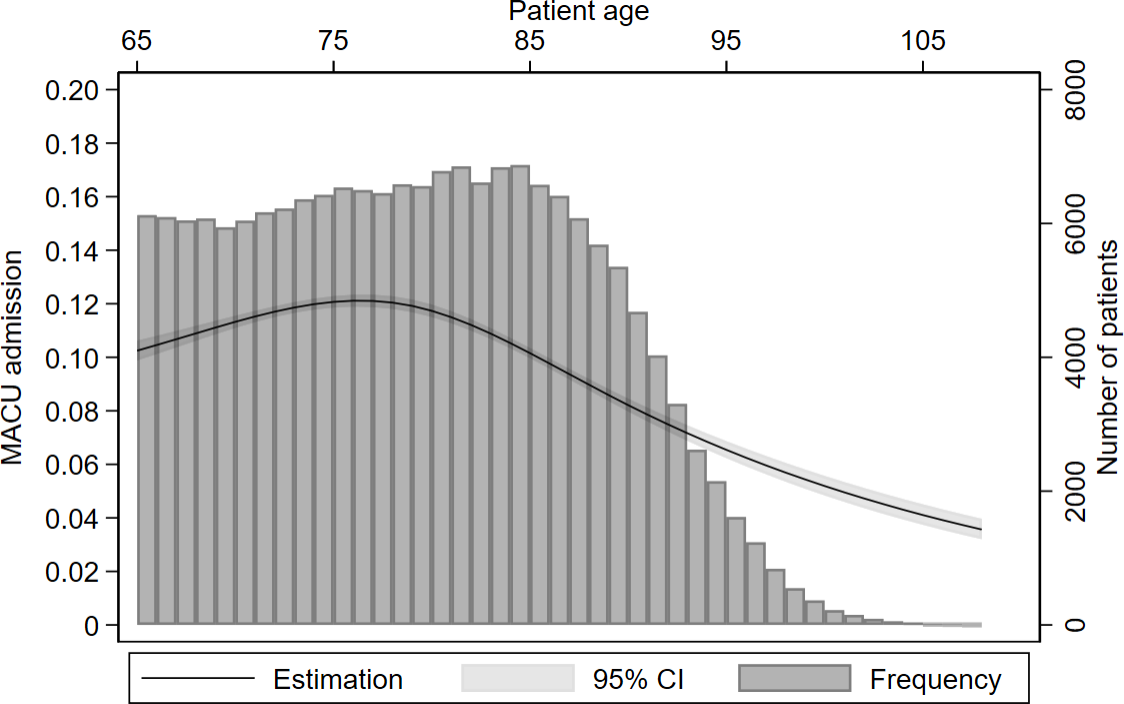
Association between age and monitored acute care unit (MACU) admissions among older patients. Older patients are less likely to be admitted to a MACU, with an inflection around 80 to 85 years old. In our multivariable analysis, younger age was identified as an independent predictor of MACU admission (75th vs 25th percentile, adjusted odds ratio 0.73, 95% CI 0.69-0.76).

Patients admitted to monitored acute care units were more frequently male, younger, and more often in a relationship (Table 1). Figure 3 shows the unadjusted association between age and monitored acute care unit admission among older patients. Patients admitted to a monitored acute care unit more frequently arrived by ambulance. They also had a higher triage level, with only 2817 (14.8%) of monitored acute care unit admissions having a lower triage level (3 or 4). In our multivariable analysis, younger age, male sex, marital status, absence of nursing home residence, arrival by ambulance, and higher triage level were all identified as independent predictors of monitored acute care unit admission (Table 3). A sensitivity analysis using age as a categorical variable did not significantly change these findings (Multimedia Appendix 2)

**Table 3.** Predictors for monitored acute care unit admission.

	Multivariable model, adjusted OR^a^ (95% CI)
Age (y)	
75th versus 25th percentile	0.73 (0.69-0.76)
Sex	
Female	Ref.^b^
Male	1.21 (1.14-1.28)
Marital status	
Married or in a relationship	Ref.
Single, divorced, or widowed	0.88 (0.83-0.93)
Nursing home resident	
No	Ref.
Yes	0.71 (0.63-0.80)
Primary language	
French	Ref.
Italian	0.83 (0.74-0.92)
Spanish	0.76 (0.66-0.89)
German	0.97 (0.83-1.14)
English	0.94 (0.78-1.15)
Portuguese	0.81 (0.65-1.02)
Others	0.78 (0.68-0.89)
Arrival by ambulance	
No	Ref.
Yes	1.87 (1.75-1.99)
Triage scale	
1: vital emergency	39.95 (25.10-63.58)
2: urgent	9.14 (5.76-14.51)
3: mild urgent	2.54 (1.60-4.03)
4: nonurgent	Ref.
Triage motive category	
Cardiology-pneumology	Ref.
Neurology-psychiatry	1.36 (1.28-1.45)
Traumatology	0.61 (0.55-0.67)
Digestive-OB/GYN^c^	1.28 (1.15-1.43)
Urology-nephrology	0.69 (0.56-0.84)
Rheumatology	0.39 (0.28-0.55)
Infectious disease	0.69 (0.58-0.83)
Dermatology	0.57 (0.43-0.75)
ENT^d^	0.35 (0.25-0.49)
Others	1.26 (1.12-1.42)

a OR: odds ratio.

b Ref.: reference.

c OB/GYN: obstetrics and gynecology.

d ENT: ear, nose, and throat.

### Outcomes

**Figure 4.**
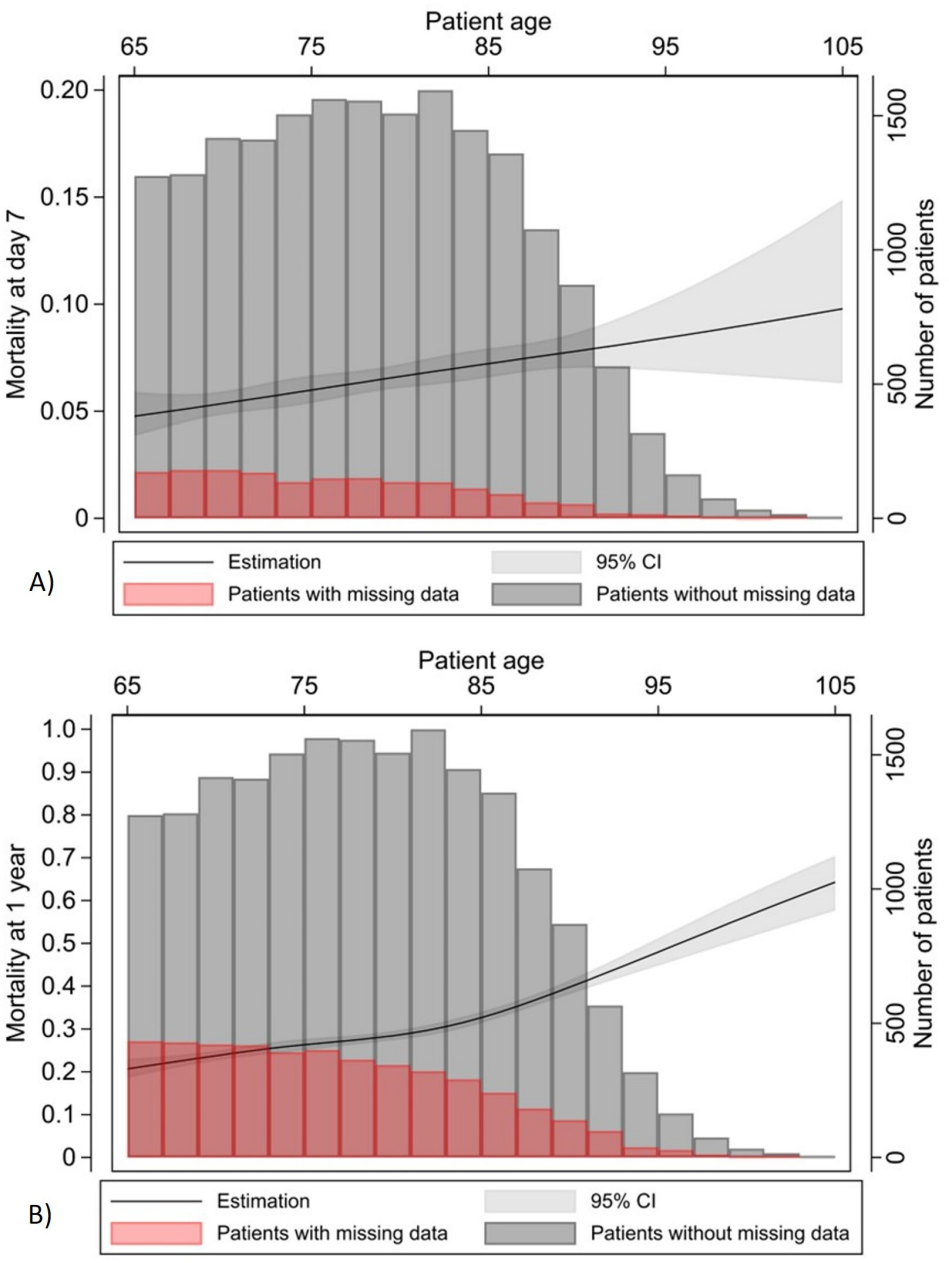
Association between age and (A) mortality at day 7 and (B) mortality at 1 year. For patients aged ≥65 years or older with monitored acute care unit admission, there was a significant association between age and mortality at day 7 (adjusted odds ratio 1.55, 95% CI 1.14-2.10) and at 1 year (adjusted odds ratio 1.28, 95% CI 1.08-1.51). Seven-day mortality was 5.8% (1105 patients), whereas 1-year mortality was 22.3% (4251 patients), with 23.9% (4553 patients) of data missing for the cohort (19,039 patients).

Seven days following monitored acute care unit admission, 1105 (5.8%) patients had died, 6184 (32.5%) patients had been discharged or transferred to long-term care, 10,139 (53.3%) patients were hospitalized in a ward, and 1611 (8.5%) were still in a monitored acute care unit (Table 4). Patients initially admitted to the intensive care unit had the highest mortality (584 patients, 13.0%) and the lowest discharge rate. Only 135 (1.2%) patients admitted to an intermediate care unit required transfer to a higher level of care. The average hospital length of stay ranged from 11.2 to 13.8 days, depending on the admitting unit. One-year mortality following monitored acute care unit admission was 22.3% (4251 patients), with 23.9% (4553 patients) of data missing for the cohort. Compared to patients admitted to an intermediate care unit or the operating room, patients initially admitted to the intensive care unit had worse outcomes (Table 4). After adjustment for other variables, there was a significant association between age and mortality at day 7 (adjusted OR 1.55, 95% CI 1.14-2.10) and at 1 year (adjusted OR 1.28, 95% CI 1.08-1.51), as illustrated in Figure 4.

**Table 4.** Outcomes of patients with monitored acute care unit admission.

	All (N=19,039)	Intensive care unit (n=4499)	Intermediate care unit (n=10,835)	Operating room (n=3705)
Status at day 7, n (%)				
Deceased	1105 (5.8)	584 (13.0)	334 (3.1)	187 (5.0)
Intensive care unit	931 (4.9)	560 (12.4)	135 (1.2)	236 (6.4)
Intermediate care unit	680 (3.6)	226 (5.0)	383 (3.5)	71 (1.9)
Ward	10,139 (53.3)	2203 (49)	6173 (57)	1763 (47.6)
Discharged or transferred	6184 (32.5)	926 (20.6)	3810 (35.2)	1448 (39.1)
Hospital length of stay (d), median (IQR)	9.0 (4.9-15.4)	10.5 (5.2-18.0)	8.9 (5.0-14.2)	8.0 (3.9-15.7)
Status at 1 year, n (%)				
Alive	10,230 (53.7)	2164 (48.1)	6096 (56.3)	1970 (53.2)
Deceased	4251 (22.3)	1355 (30.1)	2133 (19.7)	763 (20.6)
Unknown	4553 (23.9)	980 (21.8)	2606 (24.1)	972 (26.2)

## Discussion

### Principal Findings

This retrospective cohort study, spanning from 2009 to 2019, revealed a significant increase in monitored acute care unit admissions among older patients. Key predictors of monitored acute care unit admission, such as age, sex, and arrival by ambulance, were identified. While short-term outcomes were relatively good with a 7-day mortality rate of approximately 5%, long-term outcomes were poorer, with more than 20% mortality at 1 year.

Over the study period, monitored acute care unit admissions increased by 62%. This increase was not uniform and can be primarily attributed to the growing number of older patients presenting to the ED with a more modest rise in the proportion of these patients being admitted to a monitored acute care unit, from 10.3% (1379 patients) in 2009 to 11.1% (2240 patients) in 2019. Notably, this overall increase in admissions was primarily driven by a substantial rise in intermediate care unit admissions. Although changes in unit capacity over time could not be assessed, the increase in intermediate care unit admissions and the decrease in intensive care unit admissions are likely to be related. This trend may reflect a redistribution of patients and the development of new competencies within the intermediate care units. Such units can also be seen as a more tailored approach to managing older patients, providing an intermediate level of care that is perhaps more suitable for the needs of this population. Additionally, these units benefit from economic advantages, supporting their use as a cost-effective strategy to prioritize health care resource allocation.

Intensive care unit admission rates among older patients have been extensively studied, with the available literature reporting considerable variability. Some studies have found annual increases of up to 5.6% in patients over 80 years old [15], whereas others have found no significant change [16]. In contrast, data on intermediate care unit admissions are more limited, likely due to the wide variety of formats of such units (including intermediate care units, step-down units, and high dependency units), making it challenging to standardize the data [17-19]. The observed rise in intermediate care unit admissions in our study is closely linked to the overall increase in ED visits by older patients, which has directly led to higher demand for monitored care. Beyond this, several other factors may have contributed, including the growing prevalence of chronic diseases among older patients that require a level of care beyond general ward care but less than intensive care unit, along with advances in medical technologies, interventions, protocols, and admission criteria that allow for safer and more effective management of these patients in intermediate care units [20-24].

As anticipated, older patients were less likely to be admitted to a monitored acute care unit, with a notable decrease in admissions and an increase in mortality observed around the age of 85 years. This age threshold may be due to higher comorbidity rates and the presence of advanced care directives in this patient population [25,26]. Age is known to be associated with both a decrease in intensive care unit referral by emergency physicians and an increase in admission denial by intensive care physicians [27]. It might make sense, as intensive care unit admission for patients aged older than 80 years has not been shown to affect 2-year survival rates [28]. However, critical care societies recommend that decisions regarding monitored acute care unit admissions should be based on illness severity, comorbidities, and baseline functional status rather than age alone [29]. It is crucial for physicians to engage in discussions with patients and their next of kin about the potential implications of a monitored acute care unit admission, although the final decision rests with the receiving physician [30].

Our study also identified other predictors of a monitored acute care unit admission. Arrival by ambulance, which is up to 4.6 times more frequent for older patients [31] and serves as an indicator of higher clinical severity, was a strong predictor of monitored acute care unit admission. Nursing home residents were less likely to be admitted to a monitored acute care unit, likely due to higher levels of comorbidities and frailty levels, often associated with a diminished quality of life and cognitive impairment [32-34]. Neurology-psychiatry–related triage motives were the strongest predictors of monitored acute care unit admission, likely due to conditions such as strokes, which typically require a monitored acute care unit–level care. Interestingly, the French language, male sex, and marital status were also associated with monitored acute care unit admission. Language barriers are well documented as contributing to worse health outcomes, including longer hospital length of stay, higher readmission rates, and limited access to health care systems, regardless of socioeconomic status [35,36]. While female patients were predominant in the ED, they were 21% less likely to be admitted to a monitored acute care unit compared to male patients, a finding consistent with existing literature [37-39]. This disparity may be influenced by more comorbidities in men, and the tendency for female patients to set medical limitations and have advanced directives, especially when divorced or widowed [40]. However, sociocultural factors and implicit or explicit biases may also contribute to this disparity. Unmarried patients often presented with more severe illness at admission [41], possibly due to delayed medical intervention, whereas patients with a partner are more likely to receive high-level care to meet family expectations. Physicians must be aware of such disparities and strive to mitigate them in their practice.

Short-term outcomes for patients admitted to a monitored acute care unit are quite encouraging, with one-third of patients discharged and more than half transferred to the ward by day 7. However, long-term outcomes were less favorable, particularly for patients admitted to the intensive care unit, who had a 1-year mortality rate exceeding 30%. While intensive care unit mortality in older patients is well documented [42], outcomes for intermediate care unit patients have been less studied, especially among ED patients. Torres et al [43] reported no significant difference between demographics regarding in-hospital mortality after intermediate care unit admission and a 34% mortality after 2 years for patients aged older than 65 years, significantly higher than the 10% for younger patients. In a secondary analysis of the ICE-CUB2 trial focusing on ED patients, Thietart et al [44] reported 6-month mortality rates of 44% and 31% for intensive care unit and intermediate care unit patients, respectively, which were higher than those reported in other studies, including this one, which the severity of the patients can explain, as they did not include uncomplicated acute coronary syndromes and strokes. Finally, D’Andrea et al [45] found a 43% 1-year mortality rate in patients aged older than 75 years admitted to a geriatric intermediate care unit, with a significant association between age and 1-year mortality.

### Clinical and Scientific Implications

This study highlights the need to adapt health care structures to meet the growing demands of an aging population. Developing specialized geriatric acute care units or intensive care units, along with training physicians specifically to address the needs of older patients, could help raise awareness of these needs while addressing potential biases related to gender, language, and marital status. Further research should explore patient and family satisfaction and the alignment between care provided and patient wishes. Previous studies have shown that quality of life in older intensive care unit survivors is initially worse [46] but tends to improve within a year, with many older patients willing to undergo intensive care unit admission again if necessary [47,48]. Further studies could focus on developing clinical guidelines or tools specifically tailored to older patients to aid in the shared decision-making process regarding monitored acute care unit admission. These guidelines should incorporate measures such as the Functional Independence Measure or the Clinical Frailty Scale, which have been shown to correlate with resource use and outcomes [45,49,50].

### Strengths and Limitations

Some strengths and limitations need to be acknowledged. The study analyzes a large cohort over a decade, providing a robust dataset for analysis. The innovative and comprehensive approach of considering all monitored acute care unit admissions offers a broad view of high-cost care, which has not been extensively studied. Additionally, we used a rigorous statistical plan, including restricted cubic spline models to account for the nonlinear relationship between age and outcomes, to enhance the robustness of our results. The primary limitations of this study are related to the design. As a retrospective cohort, the study is prone to bias and missing data, notably the variation in monitored acute care unit beds per unit over the years, which may influence patient disposition based on unit saturation. The second major limitation is the lack of information on comorbidities, place of residence (rural vs urban), and polypharmacy. These variables are either not available as structured data in our electronic health record or are at high risk of bias. To preserve high data quality, we chose not to report them. The authors acknowledge this as a limitation of the study, with a risk of residual confounding. Results involving mortality should be considered cautiously, as mortality data were missing for 1 in 4 patients. Our study is monocentric; however, its findings are likely generalizable to other university hospitals in Switzerland and Europe, given the similar monitored acute care unit admission criteria. The exclusion of private hospitals with minimal monitored acute care unit capacity is a minor limitation, as their patients tend to be younger than 65 years. The change in the slope of the association between age and monitored acute care unit admission, as well as age and 1-year mortality, may be driven by only a small number of patients in the oldest age group, potentially distorting the true association. Finally, this study did not cover the periods of the COVID-19 pandemic, which could limit the generalizability of the results, as monitored acute care units were particularly strained during this period.

### Conclusions

This study highlights the sharp increase in monitored acute care unit admissions among older patients, reflecting the growing demand for high levels of care in this population. The rise was primarily driven by a substantial increase in intermediate care unit admissions, whereas intensive care unit admissions remained stable. These findings emphasize the need for hospitals to adapt health care infrastructure, clinical procedures, and resource allocation to meet the evolving needs of an aging population. Future work should focus on the importance of developing specialized geriatric acute care units, refining clinical guidelines, and engaging in shared decision-making to ensure equitable and effective care for all older patients.

## Supplementary material

10.2196/80629 Multimedia Appendix 1 Proportion of ED visits by older patients. Between 2009 and 2019, a total of 701,838 ED visits were recorded, with a global increase of 34.1% (56,944-76,368) in all ED visits. This increase was more pronounced for patients aged ≥65 years (14,705-22,955, +56.1%) than for patients aged <65 years (42,239 to 53,413, +26.5%; *P*<.001). ED: emergency department.

10.2196/80629 Multimedia Appendix 2 Potential predictors for MACU admission with age in categories. MACU: monitored acute care unit.

10.2196/80629 Multimedia Appendix 3 Dataset for publication. To ensure patient confidentiality, the dataset prepared for publication excludes the patient’s first language, exact admission date, and identifying information, such as patient ID.

## Abbreviations

ED: emergency department
HUG: Hôpitaux Universitaires de Genève/Geneva University Hospitals
OR: odds ratio
SMD: standardized mean difference
