# Supplementary figures and images for: Trends, Predictors, and Outcomes of Monitored Acute Care Unit Admissions in Older Adults: 10-Year Retrospective Analysis
Source: Interact J Med Res. 2026 Feb 2;15:e80629. doi: 10.2196/80629 (PMC12863243; doi:10.2196/80629)

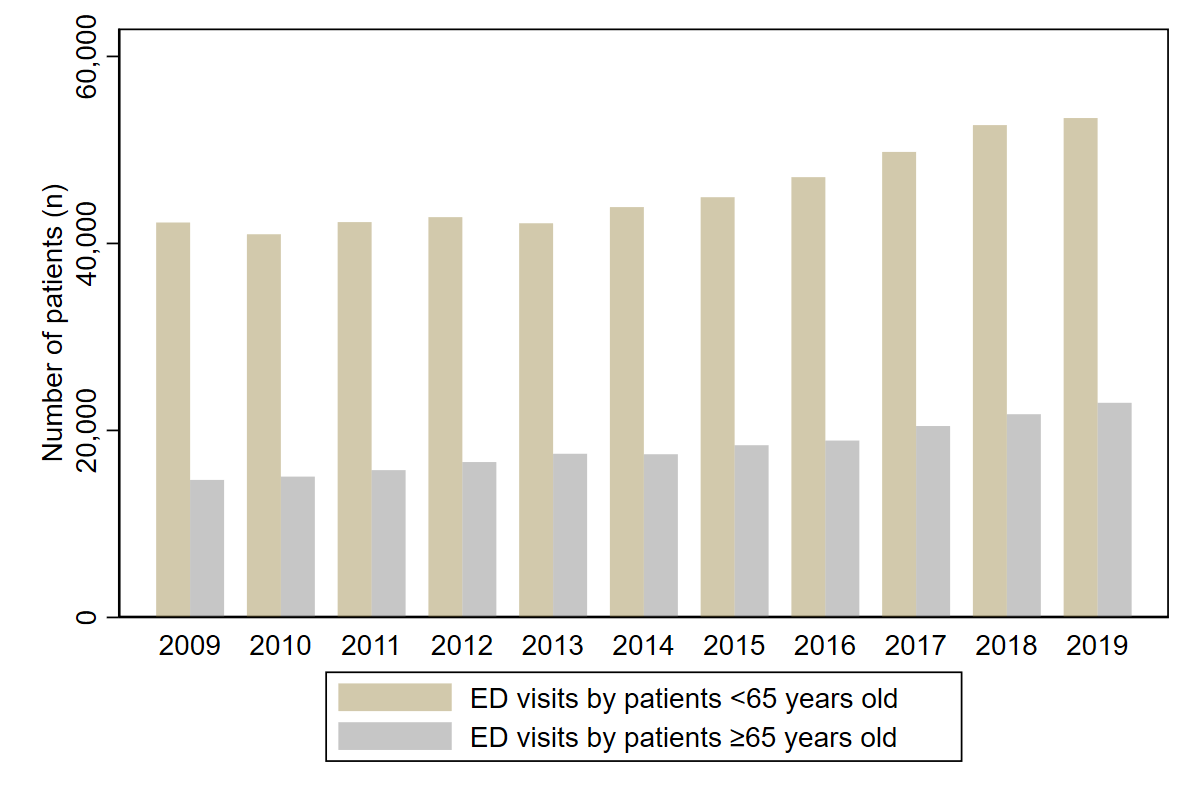

Supplement: Multimedia Appendix 1 [file ijmr-v15-e80629-s001.png]
