# Supplementary material for: Trends, Predictors, and Outcomes of Monitored Acute Care Unit Admissions in Older Adults: 10-Year Retrospective Analysis
Source: Interact J Med Res. 2026 Feb 2;15:e80629. doi: 10.2196/80629 (PMC12863243; doi:10.2196/80629)
Supplement: Multimedia Appendix 2 [file ijmr-v15-e80629-s002.docx]

## Table S1 – Potential predictors for MACU admission with age in categories.

|  | Multivariable model | |
| --- | --- | --- |
|  | Adjusted OR | 95% CI |
| Sex  Female  Male | Ref.  1.21 | 1.14-1.28 |
| Age  65-74  75-84  85-94  ≥95 | Ref.  0.88  0.66  0.42 | 0.82-0.93  0.61-0.71  0.35-0.50 |
| Marital status  Married or in a relationship  Single / Divorced / Widowed | Ref.  0.87 | 0.82-0.93 |
| Nursing home resident  No  Yes | Ref.  0.70 | 0.62-0.78 |
| Primary language  French  Italian  Spanish  German  English  Portugese  Others | Ref.  0.83  0.77  0.98  0.95  0.82  0.78 | 0.75-0.93  0.66-0.89  0.83-1.15  0.78-1.15  0.65-1.03  0.68-0.89 |
| Arrival by ambulance  No  Yes | Ref.  1.85 | 1.74-1.97 |
| Triage scale level  1  2  3  4 | 39.99  9.14  2.54  Ref. | 25.13-63.63  5.76-14.51  1.60-4.03 |
| Triage motive category  Cardiology-Pneumology  Neurology-Psychiatry  Traumatology  Digestif-Gynecology  Urology-Nephrologiy  Rhumatology  Infectious Disease  Dermatology  ENT  Others | Ref.  1.37  0.61  1.28  0.69  0.39  0.69  0.57  0.35  1.26 | 1.28-1.46  0.55-0.67  1.15-1.43  0.56-0.85  0.28-0.55  0.58-0.83  0.43-0.75  0.25-0.49  1.12-1.42 |
